# Retraction: A freight integer linear programming model under fog computing and its application in the optimization of vehicle networking deployment

**DOI:** 10.1371/journal.pone.0319590

**Published:** 2025-03-05

**Authors:** The PLOS ONE Editors

The *PLOS One* Editors retract this article [1] because it was identified as one of a series of submissions for which we have concerns about peer review integrity and potential manipulation of the publication process. These concerns call into question the validity and provenance of the reported results. We regret that the issues were not identified prior to the article’s publication.

All authors either did not respond directly or could not be reached.

## References

[1] Wang X, Qiu P. A freight integer linear programming model under fog computing and its application in the optimization of vehicle networking deployment. PLoS One. 2020;15(9):e0239628. doi: 10.1371/journal.pone.0239628 32970755 PMC7514103

